# Global and Local Concerns: What Attitudes and Beliefs Motivate Farmers to Mitigate and Adapt to Climate Change?

**DOI:** 10.1371/journal.pone.0052882

**Published:** 2012-12-26

**Authors:** Van R. Haden, Meredith T. Niles, Mark Lubell, Joshua Perlman, Louise E. Jackson

**Affiliations:** 1 Agricultural Sustainability Institute, University of California Davis, Davis, California, United States of America; 2 Department of Environmental Science and Policy, University of California Davis, Davis, California, United States of America; 3 Department of Land, Air and Water Resources, University of California Davis, Davis, California, United States of America; University of Western Australia, Australia

## Abstract

In response to agriculture's vulnerability and contribution to climate change, many governments are developing initiatives that promote the adoption of mitigation and adaptation practices among farmers. Since most climate policies affecting agriculture rely on voluntary efforts by individual farmers, success requires a sound understanding of the factors that motivate farmers to change practices. Recent evidence suggests that past experience with the effects of climate change and the psychological distance associated with people's concern for global and local impacts can influence environmental behavior. Here we surveyed farmers in a representative rural county in California's Central Valley to examine how their intention to adopt mitigation and adaptation practices is influenced by previous climate experiences and their global and local concerns about climate change. Perceived changes in water availability had significant effects on farmers' intention to adopt mitigation and adaptation strategies, which were mediated through global and local concerns respectively. This suggests that mitigation is largely motivated by psychologically distant concerns and beliefs about climate change, while adaptation is driven by psychologically proximate concerns for local impacts. This match between attitudes and behaviors according to the psychological distance at which they are cognitively construed indicates that policy and outreach initiatives may benefit by framing climate impacts and behavioral goals concordantly; either in a global context for mitigation or a local context for adaptation.

## Introduction

Even if the most optimistic emissions mitigation targets set by the Intergovernmental Panel on Climate Change are achieved, climate change will continue to progress for many decades to come [1], [2]. Given agriculture's reliance on natural resources and weather, it is inherently vulnerable to climate change impacts [3], [4]. Agriculture is also an important source of greenhouse gas emissions, accounting for 10–12% of total anthropogenic emissions annually [5]. These facts highlight the need to balance effective mitigation efforts that reduce greenhouse gas emissions with robust adaptation initiatives that enable farmers to cope with the effects of climate change and thus safeguard the resilience of social-ecological systems like agriculture [6]–[8]. In the United States, California has been one of the first states to provide a policy framework for climate change mitigation and adaptation initiatives, many of which have implications for the agricultural sector [9], [10]. Under California's Global Warming Solutions Act (AB-32), which aims to reduce greenhouse gas emissions to 1990 levels by 2020, the state is developing policies to encourage voluntary mitigation and adaptation among farmers through the adoption of water and crop management practices, renewable energy technologies, and possible participation in carbon markets [10], [11]. While a few countries now regulate emissions from agriculture through mandatory reporting, emission caps, or taxes on inputs, most countries employ a voluntary approach [11], [12]. Since these climate policies rely on bottom up voluntary efforts by rural communities and individual farmers, their success will require a sound understanding of what motivates farmers to adopt practices that facilitate mitigation and adaptation [13]–[15]. This study examines how past climate perceptions and local and global climate change beliefs and concerns influence the adoption of both mitigation and adaptation practices among farmers.

One of the primary challenges of climate change is that the risks are often perceived as being rather distant and diffused over space and time. This “psychological distance” associated with climate change is comprised of geographic, temporal, and social dimensions as well as the perceivers' feelings of uncertainty [16], [17]. Emerging research on psychological distance and its associated Construal Level Theory (CLT) suggests that individuals experience cognitive perceptions of climate change that can be either close or distant [17], [18]. For instance, climate impacts that are psychologically close (e.g. geographically or temporally proximate) are construed as concrete, tangible events relevant to the perceiver's specific local or personal context (i.e. low level construal). In contrast, climate impacts that may occur further away or well into the future are perceived as being psychologically distant, and thus require higher levels of cognitive abstraction (i.e. high level construal).

As a result, some hypothesize that framing climate change in terms of local consequences may motivate action because the personal risks are psychologically close [17], [19]. Several studies have found that first-hand experience with local climate-related events can increase concern for local climate impacts, thereby increasing an individual's response to mitigate climate change [16], [20]. For example, Spence et al. found that experience with flooding increased people's concern for climate change and their willingness conserve energy [16]. Whitmarsh found a similar effect of past experience on risk perceptions and climate change response among air pollution victims, but not among flood victims [20]. Conversely, Spence et al. found that framing climate change in terms of distant impacts can influence mitigation behavior presumably by tapping into people's core values and beliefs, which also require high level abstract construal [18], [19]. This view is consistent with other studies which indicate that high level construal leads people to act in cooperative (rather than competitive) ways when addressing environmental issues and other collective action dilemmas [21], [22]. Notably, most of the studies involving psychological distance and climate change have focused on the attitudes that influence mitigation behavior, while little is known about how construal level affects adaptation behavior. Moreover, CLT has not yet been applied to agricultural decision-making and farmers' adoption of mitigation and adaptation practices in response to climate change.

Our main hypothesis is that global beliefs and concerns about climate change will have a strong influence on farmers' mitigation behavior, while psychologically proximate concerns for local climate impacts will motivate farmers' adaptation behavior. This premise is derived from recent studies which suggest that the association between attitudes and behaviors is stronger when there is a *match in construal level* [21], [22]. While the difference in construal level between distant global concerns and proximate local concerns is self-evident, an understanding of how mitigation and adaptation behaviors are cognitively construed requires a closer examination. Greenhouse gas mitigation is a collective action problem requiring global cooperation to address the causes of climate change, while adaptation appeals to a farmer's self-interest by helping them cope with specific local consequences [23], [24]. This distinction is important because the outcomes of a farmer's efforts to mitigate emissions are diffused globally, whereas his/her efforts to adapt to local impacts yield results that are easier to observe firsthand. Thus we contend that mitigation behaviors have a higher level of construal than adaptation behaviors and predict that the construal level of their climate change concerns will match and influence the respective behaviors.

## Results and Discussion

**Figure 1:**
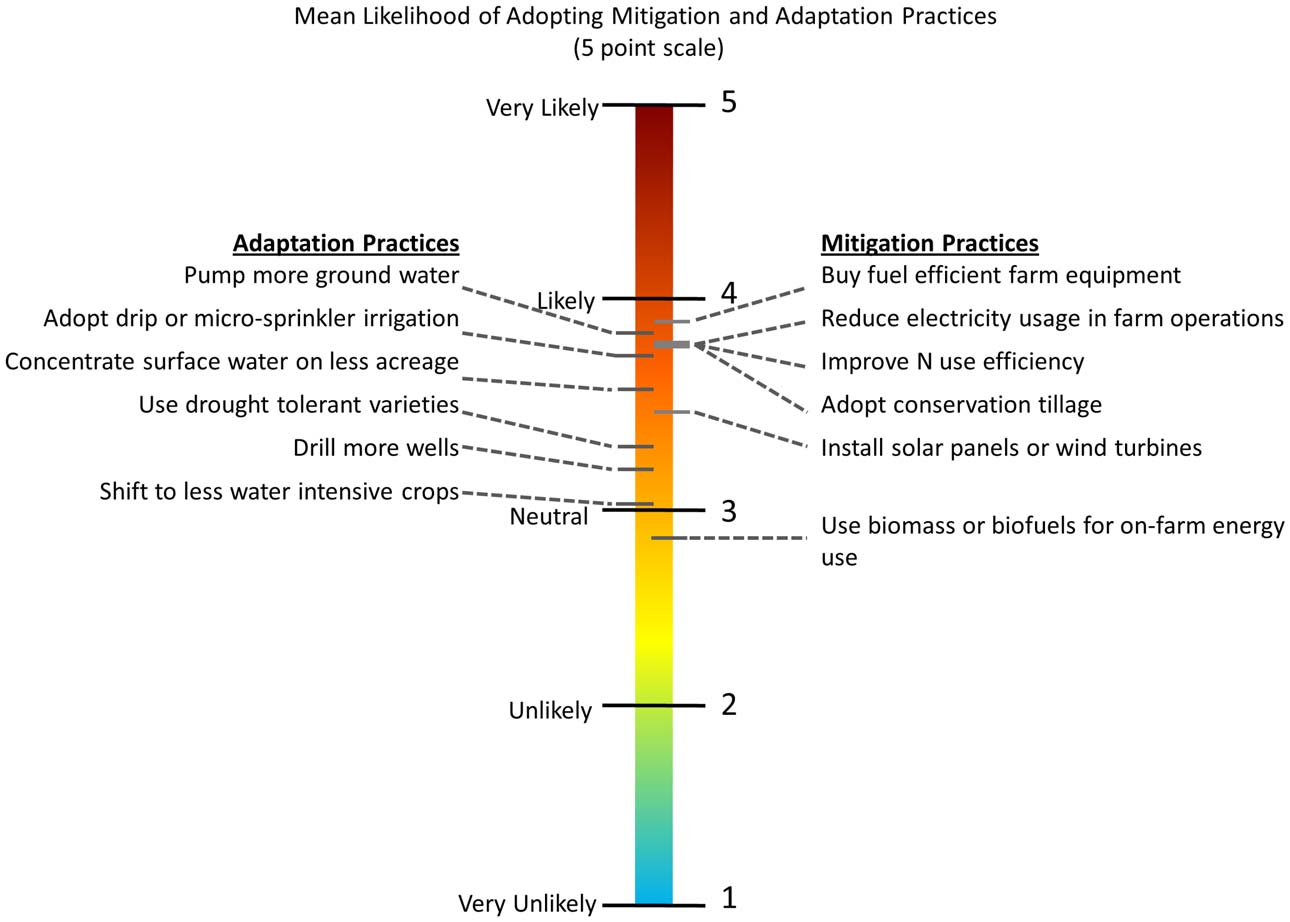
Mean likelihood of farmers adopting various mitigation and adaptation practices as measured on a 5 point scale.

To test this hypothesis we used a survey to measure farmers' past climate perceptions, local and global climate change concerns, and willingness to adopt mitigation and adaptation practices (see methods below and online supplementary material). Questions in the survey were used to develop scales which served as variables in a series of multiple-mediation models predicting farmers' intention to adopt various mitigation and adaptation practices (Table 1). Multiple mediation models assess whether the effects of an independent variable on a dependent variable are “mediated” by one or more additional variables [25], [26]. The main value of multiple mediation analysis in social psychology research is that it allows one to examine mechanisms and test theories about how information, experiences, and attitudes influence behavioral intentions [27], [28]. Here, the independent variables were farmers' perceptions of past change in local water availability and summer temperature (Table 1). We considered a total of six agricultural practices for both mitigation and adaptation which are relevant to intensive agricultural systems in the dry summer climate of California's Central Valley (Figure 1). Factor analysis yielded two sets of dependent variables for mitigation behaviors (e.g. “energy and nitrogen (N) efficiency practices” and “renewable energy technologies”) and adaptation behaviors (e.g. “new irrigation practices” and “new cropping practices”) (Table 1). Mediating variables included local concern for water availability and temperature change, and global climate change belief and concern. Key farmer demographics (age, education, local origin, and full-time farmer) and farm characteristics (acres managed and organic status) were also included as covariates. Respondents who are more concerned about climate change may also report changes in past local climate more frequently. We controlled for this by allowing independent, mediator, and demographic variables to co-vary in the multiple-mediation models. Thus, the effect of any significant mediation pathway may be viewed as over and above the effects of these other factors.

**Table 1:** Survey questions, scales, mean values, standard errors and reliability coefficients (Cronbach's α) for variables used in the multiple-mediation models.

Variable	Question/Statement	Scale	Mean	Standard Error	Cronbach's α
**Perceived Change in Local Climate** (Independent)	*Local * ***water availability*** * has _______ over the course of your farming career.* *Local * ***summer temperature*** * has _______ over the course of your farming career.*	Three Point Scale1 = increased, 2 = stayed the same, 3 = decreased	2.4572.194	0.0440.045	—
**Future Local Water Availability Concerns** (Mediator)	*How concerned are you about the following climate related risks and the future impact they may have on your farming operations during your career?*	Four Point Scale1 = Not Concerned4 = Very Concerned			
	• Less reliable surface water supply		2.535	0.113	0.77
	• Less reliable ground water supply		2.547	0.100	
	• More severe droughts		2.340	0.096	
**Future Local Temperature Concerns** (Mediator)	*How concerned are you about the following climate related risks and the future impact they may have on your farming operations during your career?*	Four Point Scale1 = Not Concerned4 = Very Concerned			
	• Fewer winter chill hours		1.659	0.090	0.86
	• Warmer summer temperatures		1.868	0.084	
	• More frequent heat waves		1.907	0.083	
**Global Climate Change Belief and Concerns** (Mediator)	*Indicate your level of agreement with the following statements*	Five Point Scale1 = Strongly Disagree5 = Strongly Agree			
	• The global climate is changing		3.414	0.113	0.93
	• Average global temperatures are increasing		3.068	0.116	
	• Human activities such as fossil fuel combustion are an important cause of climate change		3.000	0.114	
	• Climate change poses risks to agriculture globally		3.470	0.115	
	• Climate change presents more risks than benefits to agriculture globally		3.256	0.102	
**Adaptation 1 New Irrigation Practices** (Dependent)	*What is the likelihood that you would use the following management strategies, above and beyond what you currently use in a normal rainfall year?*	Five Point Scale1 = Very Unlikely5 = Very Likely			
	• Pump more ground water		3.810	0.126	0.74
	• Adopt drip or micro-sprinkler irrigation		3.684	0.137	
	• Drill more wells or seek alternative water sources		3.266	0.137	
**Adaptation 2 New Cropping Practices** (Dependent)	*What is the likelihood that you would use the following management strategies, above and beyond what you currently use in a normal rainfall year?*	Five Point Scale1 = Very Unlikely5 = Very Likely			
	• Concentrate surface water allocation on a smaller percentage of acreage		3.570	0.127	0.70
	• Use drought tolerant varieties of the crops already grown		3.367	0.131	
	• Change to a less water intensive crop		3.038	0.134	
**Mitigation 1 Energy and N Efficiency Practices** (Dependent)	*Which of the following practices would you be likely to adopt voluntarily to reduce your energy use and/or greenhouse gas emissions?*	Five Point Scale1 = Very Unlikely5 = Very Likely			
	• Invest in more fuel efficient farm equipment		3.872	0.099	0.74
	• Take measures to reduce electricity usage in farm operations or buildings		3.735	0.100	
	• Improve N use efficiency through precision placement or timing		3.735	0.072	
	• Use conservation tillage		3.701	0.092	
**Mitigation 2 Renewable Energy Technologies** (Dependent)	*Which of the following practices would you be likely to adopt voluntarily to reduce your energy use and/or greenhouse gas emissions?*	Five Point Scale1 = Very Unlikely5 = Very Likely			
	• Install solar panels or wind turbines for on-farm energy needs		3.444	0.117	0.71
	• Use biomass or biofuels for on-farm energy needs		2.830	0.104	

Independent variables for perceived change in local climate (i.e. water availability and summer temperature) are based on individual questions, while scales for the mediator and dependent variables are comprised of multiple questions that have a high reliability coefficient (Cronbach's α≥0.70).

On average, farmers in this region of California perceived a decrease in both local water availability and summer temperature over the course of their career (Table 1). When asked to consider future local climate impacts, a majority of farmers were either concerned or very concerned about less reliable ground water (57%) and surface water (56%), while 36% were concerned about more severe drought. A minority of respondents expressed concern for more frequent heat waves (27%), warmer summer temperatures (26%), or fewer winter chill hours (26%). Overall, farmers tended to show greater concern for future changes in local water availability relative to local temperature. While a majority of farmers agreed to some extent that the global climate is changing (54.4%) and poses risks to agriculture globally (53.4%), they were more divided in their views regarding whether global temperatures are increasing (37.5% agreed, 31.0% disagreed, 24.8% neutral, 5.6% uncertain) and whether human activities play a role in causing climate change (35.2% agreed, 34.5% disagreed, 26.0% neutral, 4.3% uncertain).

**Figure 2:**
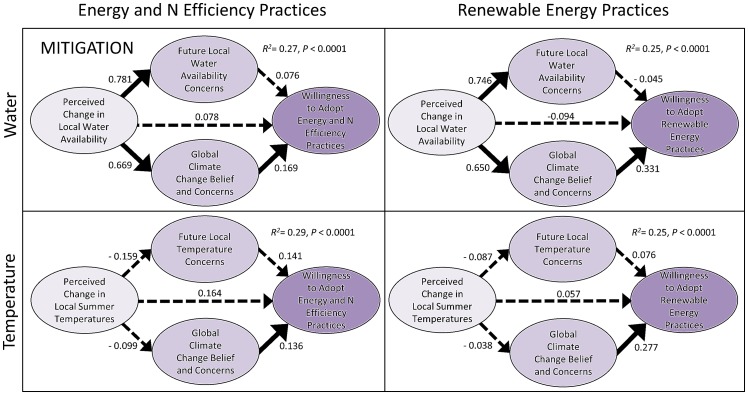
Multiple-mediation models examining the direct and indirect effects of perceived change in local climate (water, temperature) on farmers' willingness to adopt climate change mitigation practices. Values provided are unstandardized *b* coefficients indicating the strength of the relationship between variables. Solid arrows represent a significant effect between variables in the pathway (P≤0.05), while broken arrows indicate no significant effect. Overall R^2^ and P values associated with prediction of dependent variables are listed for each model.

**Figure 3:**
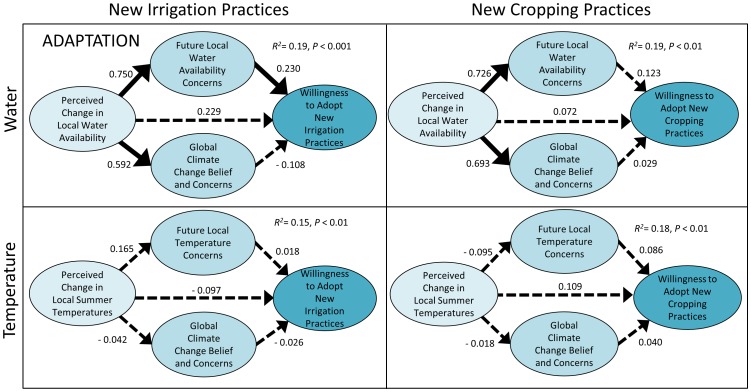
Multiple-mediation models examining the direct and indirect effects of perceived change in local climate (water, temperature) on farmers' willingness to adopt climate change adaptation practices. Values provided are unstandardized *b* coefficients indicating the strength of the relationship between variables. Solid arrows represent a significant effect between variables in the pathway (P<0.05), while broken arrows indicate no significant effect. Overall R^2^ and P values associated with prediction of dependent variables are listed for each model.

The multiple-mediation models also indicate that a perceived decrease in past water availability increased farmers' concern for local water availability in the future, and to a lesser extent, their concern for and belief in global climate change (Fig. 2, 3). In contrast, perceived changes in summer temperature had no effect on concerns for local temperature-related impacts or their belief in global climate change in any of the models (Fig. 2, 3). This lack of concern for changes in temperature is likely due to the perception among most farmers (61.9%) that no change in summer temperatures had occurred over the course of their career. Of those who did observe a change, most felt that summer temperatures had decreased (21.3%) rather than an increased (5.6%). These differences in perception may be specific to the local context since declining water availability is a persistent issue of personal and political apprehension among California farmers, while local temperatures during the summer growing season are perceived to have changed little in this region. In regions where temperature increases during the main growing season are more prominent temperature-related impacts are likely to be a more important source of concern, as has been demonstrated among African and Andean farmers [4], [29].

Consistent with our main hypothesis, the multiple-mediation analysis indicated that perceived change in past water availability had a significant indirect effect on both sets of mitigation practices, which were mediated only through farmers' global climate change beliefs and concerns. A significant direct effect of global climate change belief and concern on farmers' willingness to adopt mitigation practices was observed in all models (Fig. 2). This contrasts with adaptation practices that show a different pattern, whereby local concern for future water availability was the only significant mediator between the independent and dependent variables (Fig. 3). Among the two types of adaptation practices, only new irrigation practices were significantly affected by local water concerns, which mediated the effect of perceived change in past water availability. Adopting new cropping practices such as using a drought tolerant variety of a farmers' current crop or shifting to a less water intensive crop had a lesser likelihood of adoption among farmers (Fig. 1), which explains why these practices were not influenced by local and global concerns in our models.

These findings provide evidence that the attitudes motivating mitigation versus adaptation behavior tend to be cognitively represented at different construal levels. These results are consistent with psychological experiments conducted by Sanna et al. showing that high level construal leads to cooperative environmental behavior (e.g. mitigation practices), while lower level construal generally encourages action to safeguard one's self-interest (e.g. adaptation) [22]. The fact that psychologically distant concerns were a key determinant of mitigation behavior is likely a function of the abstract processing required for one to develop cogent beliefs (or skepticism) regarding the veracity, cause, and solution for global climate change. This suggests that adoption of mitigation practices is motivated more by a farmer's belief in and concern for long-term risks to society at large as opposed to the near-term personal risks, which, by contrast, are one of the goals of adaptation. Thus, framing climate change in terms of global impacts and the societal “gains” that might be achieved through mitigation can appeal to an individual's desire to contribute to the public good and may yield greater adoption than messages intended to provoke fear of local and/or personal consequences [19].

By contrast, adaptation among these farmers is primarily motivated by their concern for local climate impacts, which have low level construal and are by definition psychologically close (Table 1). Individuals who are operating in a psychologically proximate mindset - be they farmers or otherwise - will tend to pursue specific goals that they perceive as being both feasible and effective for dealing with problems near at hand [30]. Past studies also indicate that the adoption of agricultural practices to cope with climate change is strongly influenced by affect and emotion, presumably because affect-driven concerns tend to be construed as psychologically closer to one's personal circumstances [24], [31]. For example, when people know from past experience that certain circumstances pose a threat to them, feelings of concern and worry motivate them to take specific self-protective measures [32]. This combination of context-specific goal-setting and elevated emotional engagement, which are characteristics of a low level construal, suggest that adaptation initiatives should seek to draw farmers' attention to highly specific local impacts and perhaps more importantly to the private benefits that may be secured if they take action to cope with the consequences of climate change.

Despite these findings, the temporal dimension of psychological distance remains an important barrier to both mitigation and adaptation. This is due to the strong tendency of people to discount the long-term benefits of taking immediate action on climate change as compared to the more tangible near-term costs [24], [33]. Thus, when faced with a choice among mitigation and adaptation practices farmers may generally opt for practices that offer greater private benefits attainable in the immediate future. Here, farmers indicated that they were more likely to adopt measures to reduce fuel and electricity consumption and/or improve nitrogen use efficiency, which might allow them to save money on energy and inputs in addition to reducing their greenhouse gas emissions (Fig. 1). Likewise, adaptation practices such as drip irrigation and increased use of ground water, which are relatively easy to adopt and offer clear economic incentives, were preferred over other risk reduction measures (Fig. 1). Farmers were also less inclined to implement adaptation and mitigation practices with relatively large up-front costs (e.g. drilling new wells or installing renewable energy technologies). This indicates that there are opportunities for expanding the adoption of mitigation and adaptation practices among farmers with a shorter term planning horizon by highlighting the immediate and personal benefits that might be reaped in addition to the broader societal benefits.

## Conclusions

One conclusion that may be drawn from our work is that efforts to encourage farmers to participate in voluntary climate initiatives, ought to consider framing climate impacts and behavioral goals concordantly; either in an abstract global context for mitigation or a specific local context in the case of adaptation. The strength of this approach is that people tend to pay closer attention to persuasive messages that are able to match attitudes and desired behavior according to their levels of construal [34]. But while it seems intuitive to keep mitigation and adaptation messages focused on their respective global and local spheres, emerging evidence suggests that a combination of global and local framing may prove even more effective in stimulating the adoption of sustainable behaviors [17], [19], [30]. Many agricultural practices have ramifications for both mitigation and adaptation that involve a complex mix of benefits and tradeoffs that require farmers to balance multiple economic and environmental objectives [13], [35], [36]. In some cases, a new agricultural practice may reduce GHG emissions while also minimizing economic and/or climate-related risks. For other management strategies important economic and practical drawbacks will no doubt influence agricultural decision making more than climate-related concerns. For instance, in our study practices that improve energy or N use efficiency can often reduce production costs while maintaining yields, and as a consequence may be seen by farmers as a way to simultaneously mitigate and adapt to climate change. Within the context of CLT, practices that offer clear co-benefits to one's self and society are likely to engage both psychologically proximate and distant mindsets. As such, outreach programs that allow farmers to examine the pros and cons of individual agricultural practices by framing each in a global and local context may help facilitate agricultural decisions that are well-aligned with farmers' economic goals, their past experience, and their beliefs and concerns regarding climate change. Furthermore, having farmers consider on how certain agricultural practices address both global and local concerns may even help them span the gap between good intentions and successful implementation.

## Methods

### Ethics Statement

The University of California Institutional Review Board approved the interview protocol used in the study (approval no. 201018309-1), and documented that written informed consent was ethically obtained and that the anonymity of participants' responses was maintained. A separate ethics approval was obtained from the University of California's Institutional Review Board for the mail survey protocol (approval no. 208213-1), which was returned by participants on a voluntary and anonymous basis.

### Survey Instrument and Study Area

The survey instrument used in this study was developed with input from semi-structured interviews with a cross-section of farmers in the study area and a panel of academic researchers, agricultural officials, agricultural policy organizations (i.e. local Farm Bureau), and agricultural extension advisors. In the winter and spring of 2011, the survey was distributed by mail to 572 farmers in Yolo County, California using the tailored design method [37]. A total 162 surveys were returned with sufficiently complete answers to be used in the study (Table S2). This amounted to a raw response rate of 28.3% as a proportion of the total surveys mailed out, and a final response rate of 33.2% as a proportion of the estimated number of surveys sent to eligible farmers excluding those that were returned undeliverable [38]. The online supporting information provide a comprehensive description of the interview and survey methods (Information S1). This county was chosen for its representative mix of grain, vegetable, orchard, and livestock systems used throughout California's Central Valley (Table S1). A detailed case study of the research site, which examines innovative local strategies for climate change adaptation and mitigation, is also available in the recent peer-reviewed literature [39].

### Statistical Analysis

The statistical analysis used a series multiple-mediation models to test for direct and indirect relationships between the independent, dependent, and mediating variables detailed in Table 1. The mediating and dependent variables represent socio-cognitive constructs developed using factor analysis to group highly correlated questions into a single scale with a Cronbach's α reliability coefficient ≥0.70 (Table 1). Details regarding scale development and factor analysis can be found in (Table S3). The multiple-mediation analysis was conducted according to a product-of-coefficients approach using seemingly unrelated regression [40]. A bootstrapping method was used to reconstruct the distribution for the indirect effects (e.g. data were resampled 1000 times), and thus avoid violating the assumption of normality [25]. A summary of the models' direct and indirect effects and their confidence intervals can also be found in (Table S4).

## Supporting Information

Information S1 **This document provides additional details on the study area, semi-structured interviews, survey design, and statistical analysis.**
(PDF)

Table S1 **Yolo County agricultural statistics and top 10 commodities by market value.**
(PDF)

Table S2 **Survey response rate calculations according to AAPOR methods.**
(PDF)

Table S3 **Survey questions, scales, eigenvalues, factor loadings and reliability coefficients (Cronbach's α) for variables used in the multiple-mediation models.**
(PDF)

Table S4 **Indirect and direct effect estimations for the multiple-mediation models.**
(PDF)
