# Supplementary material for: Global and Local Concerns: What Attitudes and Beliefs Motivate Farmers to Mitigate and Adapt to Climate Change?
Source: PLoS One. 2012 Dec 26;7(12):e52882. doi: 10.1371/journal.pone.0052882 (PMC3530505; doi:10.1371/journal.pone.0052882)
Supplement: Information S1 — This document provides additional details on the study area, semi-structured interviews, survey design, and statistical analysis. (PDF) [file pone.0052882.s001.pdf]

Supporting Information S1

Global and local concerns: What attitudes and beliefs motivate farmers to mitigate and adapt to climate change?

Van R. Haden, Meredith T. Niles, Mark Lubell, Joshua Perlman, and Louise E. Jackson

Study Area

The study was conducted in Yolo County, California from 2010-2011. We focus on Yolo County for several reasons. First, Yolo County is among the first rural counties in California to specifically address climate change mitigation and adaptation in their recently passed climate action plan [1]. Consequently, concerns about the impact of climate change as well as new state and local environmental policies have brought a diverse range of stakeholders into the discussion about climate change mitigation and adaptation. A detailed case study of the research site examines context-specific strategies for mitigation and adaptation [2].

Yolo County was also chosen because it has many attributes typical of the Central Valley: small towns and cities with a changing mixture of urban, suburban, and farming-based livelihoods. Yolo County has nearly 540,000 acres of agricultural land comprising 81% of the total county land area [3]. The average farm size in Yolo County is 488 acres, though nearly half of all farms are less than 50 acres [4]. The average market value of agricultural products sold per farm is approximately $390,000 a year, with an average net cash income just below $100,000 annually [4].

The agricultural landscape in Yolo County includes a mix of irrigated row crops and orchards grown on alluvial plains, rice cultivated on poorly drained soils along the western bank of the Sacramento River, and grazed rangelands on the eastern slope of California’s Coastal Range. The most significant crops in the area include tomatoes, alfalfa, wine grapes, almonds and walnuts [5]. Livestock grazed on rangeland occupy 24% of county land area [3]. Beef cattle and sheep are the main livestock types, while dairy and poultry are relatively minor components of the local livestock industry [5]. In recent years there has been a local trend towards larger farm sizes with less crop diversification [2]. Organic farms make up less than 5% of total irrigated cropland, but organic acreage has expanded considerably over the past 20 years [5]. Agricultural production in the county is largely reliant on intensive inputs including fossil fuels and synthetic

fertilizers. The mitigation and adaptation strategies examined in this study were chosen due to their local relevance, and thus may not always reflect practices suitable for other regions.

Semi-structured Interviews

Separate Institutional Review Board approvals were obtained for the interview and mail survey components of this study. To develop a sound ethnographic understanding of local farmers' livelihoods, their perceptions of climate change, and their views regarding climate risks, we conducted semi-structured interviews with eleven farmers and two agricultural extension agents in the fall of 2010. A purposive sampling strategy was used to recruit local respondents from a cross section of farm sizes, cropping systems, management practices, and market orientations [6,7]. Interviewers followed a set of open ended questions to minimize prompting and interviewer bias, but allow respondents to share personal experiences from their career in agriculture. Interviewees were asked questions related to their perceptions of climate change, climate change mitigation and adaptation practices, regulations, marketing, and perceived changes in climate in the region over time. The interviews were voluntary and respondents were given the opportunity to remain anonymous. Interviews were transcribed and analyzed using axial coding methods in N-Vivo version 9.0 (QSR International, Victoria, AUS) [8].

Survey Design

The results of the semi-structured interviews were used to design a mail survey carried out in the winter and spring of 2011. The survey instrument was developed with input from a cross-section of farmers in the study area and a panel of academic researchers, agricultural officials, agricultural policy organizations, agricultural and food industry representatives, and agricultural extension workers. A copy of the final survey is available upon request to the corresponding authors. The survey sample was drawn from a mail and phone list of 572 individuals in Yolo County who have submitted pesticide use permits to the Yolo County Agriculture Commissioner's office. The State of California requires all farms and businesses that apply federally restricted use pesticide or state restricted material (including organic pesticides) must hold an applicator certificate and register the use of the pesticide through the local agricultural commissioner [9]. The local agricultural commissioner then maintains this database as a part of

the public record. Since this database includes the vast majority of farmers in Yolo County it provides a viable list of farmers to use in this study. The mail survey was conducted using the tailored design method in order to maximize survey response rate [10]. An alert postcard was mailed in mid-February and followed a week later by a survey packet containing a cover letter, survey booklet, and return envelope. A second round of postcards and survey packets were mailed two weeks later. A final round of follow-up postcards was mailed and phone calls were made to those on the mail list that did not respond. Additional surveys were sent when requested by farmers. Response rate was calculated using methods published by the American Association for Public Opinion Research (Table S2) [11]. The follow-up phone calls indicated that approximately 28 people were outside the intended scope of our survey (e.g. managers of golf courses, grain storage facilities, university research stations). Of the 572 surveys mailed out, 162 were returned with sufficiently complete answers to be used in the study. This amounts to a raw response rate of 28.3% as a percentage of the total surveys mailed out, and a final response rate of 33.2% (Eq. 1) as a percentage of the estimated number of surveys sent to eligible farmers excluding those that were returned undeliverable[8].

Eq. 1 $$\text{Final Response Rate} = \frac{\text{Total Returned Surveys}}{(\text{Total Returned Surveys}) + \text{E}*(\text{Total Unreturned Surveys})}$$

Variable Measurement and Multiple Mediation Analysis

Multiple mediation models assess whether or not the effects of an independent variable on a dependent variable are mediated by one or more additional "mediating" variables [12]. Likewise, we hypothesized that past perceptions of water availability and temperature change may affect farmers' mitigation and adaptation behavior through their concern for local and global climate impacts, which act as mediating variables. In this study, the independent variables for perceived change in local water availability and summer temperature were based on individual questions in the survey (Table S3). The four dependent variables that characterize mitigation and adaptation behavior, and the three mediator variables that describe local and global concerns, are composite scales made up of related questions that reflect latent socio-cognitive constructs (Table S3). Factor analysis with a varimax rotation was applied to each set of questions germane to a particular latent construct in order to determine composite scales (Table S3). Only variables with

factor loadings ≥ 0.40 on at least one factor were retained for further analysis [13]. Since a single factor solution was obtained for each factor analysis, the data justifies treating the retained questions in each construct as single composite scale. Scales were created by averaging responses across the retained questions for a given construct. Cronbach's α reliability coefficient was also used to measure the internal consistency of each scale. Table S3 shows that each of the scales had an $\alpha \geq 0.70$ and thus are valid for use [14]. Important farmer demographics (age, education, local origin, and full-time farmer) and farm characteristic (acres managed and organic status) were also measured.

The multiple-mediation analysis was conducted according to a product-of-coefficients approach using seemingly unrelated regression in STATA version 11.0 (StataCorp LP, College Station, TX, USA). To conduct the multiple mediation analysis we ran seemingly unrelated regression models with bootstrapped standard error calculations (n= 1,000), which did not require assuming that indirect effects be normally distributed [13]. Farmer demographics and farm characteristics were included as covariates during each step of the regression analysis. To avoid overestimating the indirect effects, the models also allowed the mediator variables to co-vary. A total of eight multiple mediation models were produced, four for mitigation and four for adaptation (Fig. 1, Fig. 2). In three of our eight models we observed "indirect-only mediation", a term used by Zhao et al. [15] to describe mediation pathways where the indirect effects are significant and the direct effects are not ($P \leq .10$) (Table S4). Early work on mediation analysis assumed that a significant direct relationship between an independent and dependent variable must be established before possible indirect effects (i.e. mediation) can be tested for [16]. However, recent studies by Hayes [12] and Zhao et al. [15] provide empirical evidence that this assumption is flawed, and contend that a failure to test for indirect effects when no direct effect is present can often lead researchers to overlook or discard likely causal mechanisms. Thus, in light of this recent shift in mediation analysis theory, our study should be viewed as both statistically sound, and an important case in point, which suggests that future efforts to understand the effects of climate change attitudes on behavior should go beyond examining mere direct effects.

References

1. Yolo County Government (2010) Yolo County Climate Action Plan: A Strategy for Smart Growth Implementation, Greenhouse Gas Reduction, and Adaptation to Global Climate Change. Available: http://www.yolocounty.org/Index.aspx?page=2004. Accessed 15 November 2012.
2. Jackson LE, Wheeler SM, Hollander AD, O'Geen AT, Orlove BS et al. (2011) Case study on potential agricultural responses to climate change in a California landscape. Clim Change 109 (S1): S407-S427.
3. Farmland Mapping and Monitoring Program (2008) California Department of Conservation. Yolo County Important Farmland Data. Available: http://redirect.conservation.ca.gov/DLRP/fmmp/product_page.asp. Accessed 20 April 2012.
4. U.S. Department of Agriculture (2007) 2007 Census of Agriculture County Profile: Yolo County. Washington, DC: National Agricultural Statistics Service.
5. Yolo County Government (2011) 2011 Agricultural Crop Report: Available: http://www.yolocounty.org/Index.aspx?page=1419. Accessed 20 April 2012.
6. Kemper EA, Stringfield S, Teddlie T (2003) Mixed methods sampling strategies in social science research. In: Tashakkori A, Teddlie C, editors. Handbook of mixed methods in social and behavioral research. Thousand Oaks, CA: Sage Publishing Co. pp. 273-296.
7. Pearce T, Smit B, Duerden D, Ford J, Goose A, Kataoyak F et al.(2010) Inuit vulnerability and adaptive capacity to climate change in Ulukhaktok, Northwest Territories. Canada Polar Rec 46: 156–177.
8. Corbin JM, Strauss A (1990) Grounded theory research: Procedures, canons, and evaluative criteria. Qual Sociol 13: 3-21.
9. Yolo County Government (2010) Pesticide Use Enforcement Permit and Licensing Information. Available: http://www.yolocounty.org/Index.aspx?page=1103. Accessed 10 November 2012.
10. Dillman DA, Smyth JD, Christian LM (2009) Internet, Mail and Mixed Mode Surveys: The Tailored Design Method, 3rd edition, Hoboken, NJ: John Wiley & Sons Inc. 512 p.
11. American Association for Public Opinion (2011) Standard Definitions: Final Dispositions of Case Codes and Outcome Rates for Surveys. Available: http://www.aapor.org/AM/Template.cfm?Section=Standard_Definitions2&Template=/CM/ContentDisplay.cfm&ContentID=3156. Accessed 20 January 2012.
12. Hayes AF (2009) Beyond Baron and Kenny: Statistical mediation analysis in the new millennium. Commun Monogr 76: 408-420.
13. Costello AB, Osborne JW (2005) Best practices in exploratory factor analysis: Four recommendations for getting the most from your analysis. Prac Assess Res and Eval 10: 173-178.
14. Nunnally JC (1978) Pyschometric Theory. 2nd edition, New York: McGraw-Hill Co. 701 p.

15. Zhao X, Lynch JG, Chen Q (2010) Reconsidering Baron and Kenny: Myths and truths about mediation analysis. J Consum Res 37: 197-206.

16. Baron RM, Kenny DA (1986) The moderator-mediator variable distinction in social psychological research: Conceptual, strategic, and statistical considerations. J Pers Soc Psychol 51: 1173-1182.

Table S1. Yolo County agricultural statistics and top 10 commodities by market value.

**Yolo County Agriculture Statistics**	
Percent of County Land in Agriculture[1]	81%
Percent of County Land in Cropland	57%
Percent of County Land in Rangeland	24%
Average Farm Size[2]	488 acres
**Top 10 Commodities by Market Value[3]**	
1.Processing Tomatoes	6.Almonds
2.Wine Grapes	7.Organic Production
3.Rice	8.Walnuts
4.Seed Crops	9.Cattle and Calves
5.Alfalfa Hay	10.Wheat

[1] FMMP 2008
[2] USDA 2007
[3] Yolo County Government 2009

Table S2. Survey response rate calculations according to AAPOR methods[1].

Formula component	Frequencies
**Total Returned Surveys =** I + P	**162**
Complete interview (I) - Returned completed survey	158
Partial interview (P) - Returned partially completed survey with sufficient information for inclusion	4
**Total Unreturned Surveys =** NR + RU	**382**
Mail Survey Not Returned (NR)	365
Mail Survey Returned Undelivered (RU)	17
**Not eligible (NE)** - Screened out of sample population	28
	**Proportion**
**Estimated proportion of cases of unknown eligibility that are eligible (E) =** I+P/(I+P)+NE	0.853
**Raw Response Rate**	**0.283**
**Final Response Rate**	**0.332**

[1]AAPOR 2011

Table S3. Survey questions, scales, eigenvalues, factor loadings and reliability coefficients (Cronbach's α) for variables used in the multiple-mediation models. Independent variables for perceived change in local climate (i.e. water availability and summer temperature) are based on individual questions, while scales for the mediator and dependent variables are comprised of multiple questions that load on a single factor.

Variable	Statement/Question	Scale	Eigenvalue	Factor Loading	Cronbach's α
**Perceived Change in Local Climate** (Independent)	*Local* ***water availability*** *has _______ over the course of your farming career.*	Three Point Scale 1 = increased, 2 =stayed the same, 3 = decreased	---	---	---
	Local ***summer temperature*** *has _______ over the course of your farming career.*		---	---	---
**Future Local Water Availability Concerns** (Mediator)	*How concerned are you about the following climate related risks and the future impact they may have on your farming operations during your career?*	Four Point Scale 1 = Not Concerned 4 = Very Concerned	1.52		0.77
	• Less reliable surface water supply			0.64	
	• Less reliable ground water supply			0.74	
	• More severe droughts			0.75	
**Future Local Temperature Concerns** (Mediator)	*How concerned are you about the following climate related risks and the future impact they may have on your farming operations during your career?*	Four Point Scale 1 = Not Concerned 4 = Very Concerned	2.07		0.86
	• Fewer winter chill hours			0.58	
	• Warmer summer temperatures			0.93	
	• More frequent heat waves			0.91	
**Global Climate Change Belief and Concerns** (Mediator)	*Indicate your level of agreement with the following statements*	Five Point Scale 1 = Strongly Disagree 5= Strongly Agree	3.68		0.93
	• The global climate is changing			0.68	
	• Average global temperatures are increasing			0.77	
	• Human activities such as fossil fuel combustion are an important cause of climate			0.63	
	• Climate change poses risks to agriculture globally			0.54	
	• Climate change presents more risks than benefits to agriculture globally			0.41	
**Adaptation 1 New Irrigation Practices** (Dependent)	*What is the likelihood that you would use the following management strategies, above and beyond what you currently use in a normal rainfall year?*	Five Point Scale 1 = Very Unlikely 5 = Very Likely	1.41		0.74
	• Pump more ground water			0.70	
	• Drill more wells or seek alternative water sources			0.75	
	• Adopt drip or micro-sprinkler irrigation			0.59	
**Adaptation 2 New Cropping Practices** (Dependent)	*What is the likelihood that you would use the following management strategies, above and beyond what you currently use in a normal rainfall year?*	Five Point Scale 1 = Very Unlikely 5 = Very Likely	1.27		0.70
	• Use drought tolerant varieties of the crops already grown			0.72	
	• Change to a less water intensive crop			0.70	
	• Concentrate surface water allocation on a smaller percentage of acreage			0.52	
**Mitigation 1 Energy and N Efficiency Practices** (Dependent)	*Which of the following practices would you be likely to adopt voluntarily to reduce your energy use and/or greenhouse gas emissions?*	Five Point Scale 1 = Very Unlikely 5 = Very Likely	1.66		0.74
	• Take measures to reduce electricity usage in farm operations or buildings			0.59	
	• Invest in more fuel efficient farm equipment			0.68	
	• Use conservation tillage			0.72	
	• Improve N use efficiency through precision placement or timing			0.59	
**Mitigation 2 Renewable Energy Technologies** (Dependent)	*Which of the following practices would you be likely to adopt voluntarily to reduce your energy use and/or greenhouse gas emissions?*	Five Point Scale 1 = Very Unlikely 5 = Very Likely	0.84		0.71
	• Install solar panels or wind turbines for on-farm energy needs			0.65	
	• Use biomass or biofuels for on-farm energy needs			0.65	

Table S4. Indirect and direct effect estimations for the multiple-mediation models. Mediation type calculated based on P ≤ 0.10.

		Indirect Effects									Direct Effects		
Independent Variables	Dependent Variables	Local Concerns Effect	P Value	Bias Corrected 95% CI	Global Concerns Effect	P Value	Bias Corrected 95% CI	Total Indirect Effect	P Value	Bias Corrected 95% CI	Beta	P Value	Type of Mediation
Water Availability	Efficiency Mitigation Practices	0.060	0.42	-0.076, 0.213	0.113	0.06	0.022, 0.250	0.172	0.05	0.017, 0.373	0.078	0.58	Indirect-only Mediation
Water Availability	Renewable Energy Mitigation Practices	-0.034	0.76	-0.283, 0.159	0.215	0.02	0.039, 0.406	0.182	0.17	-0.070, 0.461	-0.094	0.64	Indirect-only Mediation
Summer Temperatures	Efficiency Mitigation Practices	-0.022	0.51	-0.133, 0.015	-0.013	0.64	-0.114, 0.021	-0.036	0.45	-0.160, 0.041	0.164	0.18	Non-Mediation
Summer Temperatures	Renewable Energy Mitigation Practices	-0.007	0.83	-0.125, 0.030	-0.010	0.86	-0.134, 0.120	-0.017	0.81	-0.165, 0.120	0.057	0.76	Non-Mediation
Water Availability	Irrigation Adaptation	0.173	0.06	0.017, 0.378	-0.064	0.28	-0.197, 0.038	0.109	0.25	-0.069, 0.307	0.229	0.17	Indirect-only Mediation
Water Availability	Crop Adaptation Practices	0.089	0.30	-0.073, 0.270	0.020	0.80	-0.145, 0.175	0.109	0.35	-0.114, 0.346	0.072	0.72	Non-Mediation
Summer Temperatures	Irrigation Adaptation	-0.004	0.91	-0.103, 0.040	0.001	0.95	-0.029, 0.054	-0.002	0.94	-0.086, 0.055	-0.097	0.53	Non-Mediation
Summer Temperatures	Crop Adaptation Practices	-0.008	0.77	-0.125, 0.019	-0.001	0.97	-0.061, 0.031	-0.009	0.80	-0.116, 0.040	0.109	0.55	Non-Mediation
