# Supplementary material for: Global and Local Concerns: What Attitudes and Beliefs Motivate Farmers to Mitigate and Adapt to Climate Change?
Source: PLoS One. 2012 Dec 26;7(12):e52882. doi: 10.1371/journal.pone.0052882 (PMC3530505; doi:10.1371/journal.pone.0052882)
Supplement: Table S1 — Yolo County agricultural statistics and top 10 commodities by market value. (PDF) [file pone.0052882.s002.pdf]

Table S1. Yolo County agricultural statistics and top 10 commodities by market value.

**Yolo County Agriculture Statistics**	
Percent of County Land in Agriculture[1]	81%
Percent of County Land in Cropland	57%
Percent of County Land in Rangeland	24%
Average Farm Size[2]	488 acres
**Top 10 Commodities by Market Value[3]**	
1.Processing Tomatoes	6.Almonds
2.Wine Grapes	7.Organic Production
3.Rice	8.Walnuts
4.Seed Crops	9.Cattle and Calves
5.Alfalfa Hay	10.Wheat

[1] FMMP 2008
[2] USDA 2007
[3] Yolo County Government 2009
