# Supplementary material for: Global and Local Concerns: What Attitudes and Beliefs Motivate Farmers to Mitigate and Adapt to Climate Change?
Source: PLoS One. 2012 Dec 26;7(12):e52882. doi: 10.1371/journal.pone.0052882 (PMC3530505; doi:10.1371/journal.pone.0052882)
Supplement: Table S2 — Survey response rate calculations according to AAPOR methods. (PDF) [file pone.0052882.s003.pdf]

Table S2. Survey response rate calculations according to AAPOR methods[1].

Formula component	Frequencies
**Total Returned Surveys =** I + P	**162**
Complete interview (I) - Returned completed survey	158
Partial interview (P) - Returned partially completed survey with sufficient information for inclusion	4
**Total Unreturned Surveys =** NR + RU	**382**
Mail Survey Not Returned (NR)	365
Mail Survey Returned Undelivered (RU)	17
**Not eligible (NE)** - Screened out of sample population	28
	**Proportion**
**Estimated proportion of cases of unknown eligibility that are eligible (E) =** I+P/(I+P)+NE	0.853
**Raw Response Rate**	**0.283**
**Final Response Rate**	**0.332**

[1]AAPOR 2011
