# Supplementary material for: Global and Local Concerns: What Attitudes and Beliefs Motivate Farmers to Mitigate and Adapt to Climate Change?
Source: PLoS One. 2012 Dec 26;7(12):e52882. doi: 10.1371/journal.pone.0052882 (PMC3530505; doi:10.1371/journal.pone.0052882)
Supplement: Table S3 — Survey questions, scales, eigenvalues, factor loadings and reliability coefficients (Cronbach's α) for variables used in the multiple-mediation models. (PDF) [file pone.0052882.s004.pdf]

Table S3. Survey questions, scales, eigenvalues, factor loadings and reliability coefficients (Cronbach's α) for variables used in the multiple-mediation models. Independent variables for perceived change in local climate (i.e. water availability and summer temperature) are based on individual questions, while scales for the mediator and dependent variables are comprised of multiple questions that load on a single factor.

Variable	Statement/Question	Scale	Eigenvalue	Factor Loading	Cronbach's α
**Perceived Change in Local Climate** (Independent)	*Local **water availability** has _______ over the course of your farming career.*	Three Point Scale 1 = increased, 2 =stayed the same, 3 = decreased	---	---	---
	*Local **summer temperature** has _______ over the course of your farming career.*		---	---	---
**Future Local Water Availability Concerns** (Mediator)	*How concerned are you about the following climate related risks and the future impact they may have on your farming operations during your career?*	Four Point Scale 1 = Not Concerned 4 = Very Concerned	1.52		0.77
	• Less reliable surface water supply			0.64	
	• Less reliable ground water supply			0.74	
	• More severe droughts			0.75	
**Future Local Temperature Concerns** (Mediator)	*How concerned are you about the following climate related risks and the future impact they may have on your farming operations during your career?*	Four Point Scale 1 = Not Concerned 4 = Very Concerned	2.07		0.86
	• Fewer winter chill hours			0.58	
	• Warmer summer temperatures			0.93	
	• More frequent heat waves			0.91	
**Global Climate Change Belief and Concerns** (Mediator)	*Indicate your level of agreement with the following statements*	Five Point Scale 1 = Strongly Disagree 5= Strongly Agree	3.68		0.93
	• The global climate is changing			0.68	
	• Average global temperatures are increasing			0.77	
	• Human activities such as fossil fuel combustion are an important cause of climate			0.63	
	• Climate change poses risks to agriculture globally			0.54	
	• Climate change presents more risks than benefits to agriculture globally			0.41	
**Adaptation 1 New Irrigation Practices** (Dependent)	*What is the likelihood that you would use the following management strategies, above and beyond what you currently use in a normal rainfall year?*	Five Point Scale 1 = Very Unlikely 5 = Very Likely	1.41		0.74
	• Pump more ground water			0.70	
	• Drill more wells or seek alternative water sources			0.75	
	• Adopt drip or micro-sprinkler irrigation			0.59	
**Adaptation 2 New Cropping Practices** (Dependent)	*What is the likelihood that you would use the following management strategies, above and beyond what you currently use in a normal rainfall year?*	Five Point Scale 1 = Very Unlikely 5 = Very Likely	1.27		0.70
	• Use drought tolerant varieties of the crops already grown			0.72	
	• Change to a less water intensive crop			0.70	
	• Concentrate surface water allocation on a smaller percentage of acreage			0.52	
**Mitigation 1 Energy and N Efficiency Practices** (Dependent)	*Which of the following practices would you be likely to adopt voluntarily to reduce your energy use and/or greenhouse gas emissions?*	Five Point Scale 1 = Very Unlikely 5 = Very Likely	1.66		0.74
	• Take measures to reduce electricity usage in farm operations or buildings			0.59	
	• Invest in more fuel efficient farm equipment			0.68	
	• Use conservation tillage			0.72	
	• Improve N use efficiency through precision placement or timing			0.59	
**Mitigation 2 Renewable Energy Technologies** (Dependent)	*Which of the following practices would you be likely to adopt voluntarily to reduce your energy use and/or greenhouse gas emissions?*	Five Point Scale 1 = Very Unlikely 5 = Very Likely	0.84		0.71
	• Install solar panels or wind turbines for on-farm energy needs			0.65	
	• Use biomass or biofuels for on-farm energy needs			0.65	
