# Supplementary material for: Global and Local Concerns: What Attitudes and Beliefs Motivate Farmers to Mitigate and Adapt to Climate Change?
Source: PLoS One. 2012 Dec 26;7(12):e52882. doi: 10.1371/journal.pone.0052882 (PMC3530505; doi:10.1371/journal.pone.0052882)
Supplement: Table S4 — Indirect and direct effect estimations for the multiple-mediation models. (PDF) [file pone.0052882.s005.pdf]

Table S4. Indirect and direct effect estimations for the multiple-mediation models. Mediation type calculated based on P ≤ 0.10.

		Indirect Effects									Direct Effects		
Independent Variables	Dependent Variables	Local Concerns Effect	P Value	Bias Corrected 95% CI	Global Concerns Effect	P Value	Bias Corrected 95% CI	Total Indirect Effect	P Value	Bias Corrected 95% CI	Beta	P Value	Type of Mediation
Water Availability	Efficiency Mitigation Practices	0.060	0.42	-0.076, 0.213	0.113	0.06	0.022, 0.250	0.172	0.05	0.017, 0.373	0.078	0.58	Indirect-only Mediation
Water Availability	Renewable Energy Mitigation Practices	-0.034	0.76	-0.283, 0.159	0.215	0.02	0.039, 0.406	0.182	0.17	-0.070, 0.461	-0.094	0.64	Indirect-only Mediation
Summer Temperatures	Efficiency Mitigation Practices	-0.022	0.51	-0.133, 0.015	-0.013	0.64	-0.114, 0.021	-0.036	0.45	-0.160, 0.041	0.164	0.18	Non-Mediation
Summer Temperatures	Renewable Energy Mitigation Practices	-0.007	0.83	-0.125, 0.030	-0.010	0.86	-0.134, 0.120	-0.017	0.81	-0.165, 0.120	0.057	0.76	Non-Mediation
Water Availability	Irrigation Adaptation	0.173	0.06	0.017, 0.378	-0.064	0.28	-0.197, 0.038	0.109	0.25	-0.069, 0.307	0.229	0.17	Indirect-only Mediation
Water Availability	Crop Adaptation Practices	0.089	0.30	-0.073, 0.270	0.020	0.80	-0.145, 0.175	0.109	0.35	-0.114, 0.346	0.072	0.72	Non-Mediation
Summer Temperatures	Irrigation Adaptation	-0.004	0.91	-0.103, 0.040	0.001	0.95	-0.029, 0.054	-0.002	0.94	-0.086, 0.055	-0.097	0.53	Non-Mediation
Summer Temperatures	Crop Adaptation Practices	-0.008	0.77	-0.125, 0.019	-0.001	0.97	-0.061, 0.031	-0.009	0.80	-0.116, 0.040	0.109	0.55	Non-Mediation
